# Supplementary material for: Identification of Ancestry Informative Marker (AIM) Panels to Assess Hybridisation between Feral and Domestic Sheep
Source: Animals (Basel). 2020 Mar 30;10(4):582. doi: 10.3390/ani10040582 (PMC7222383; doi:10.3390/ani10040582)

Figure S1. Unsupervised Admixture analysis for K=2 evaluated on the full set of reference populations (SAR and MSar).

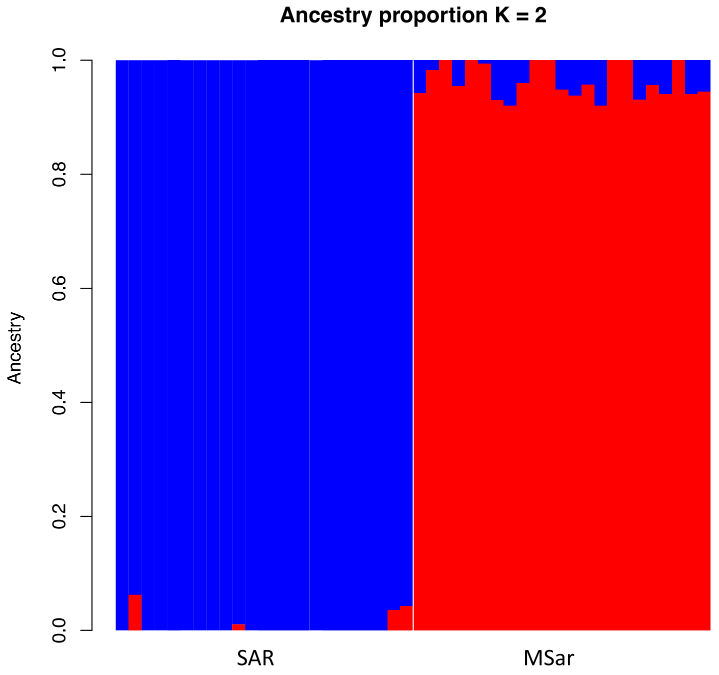

Figure S2. – Plot of PC1 loadings, squared and ordered. Red dots represent SNPs selected.

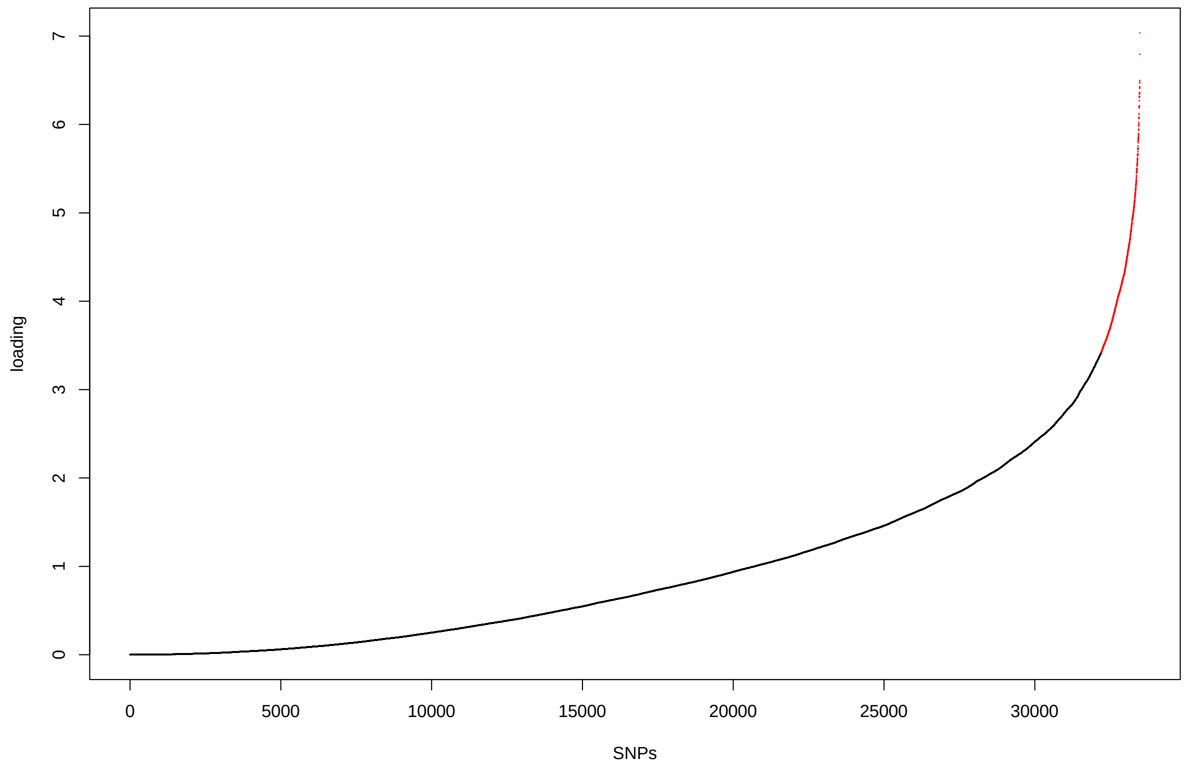

Figure S3. For each one of the 20 subsequent iterations is shown the consensus number of SNPs "confirmed important" in all the performed iterations.

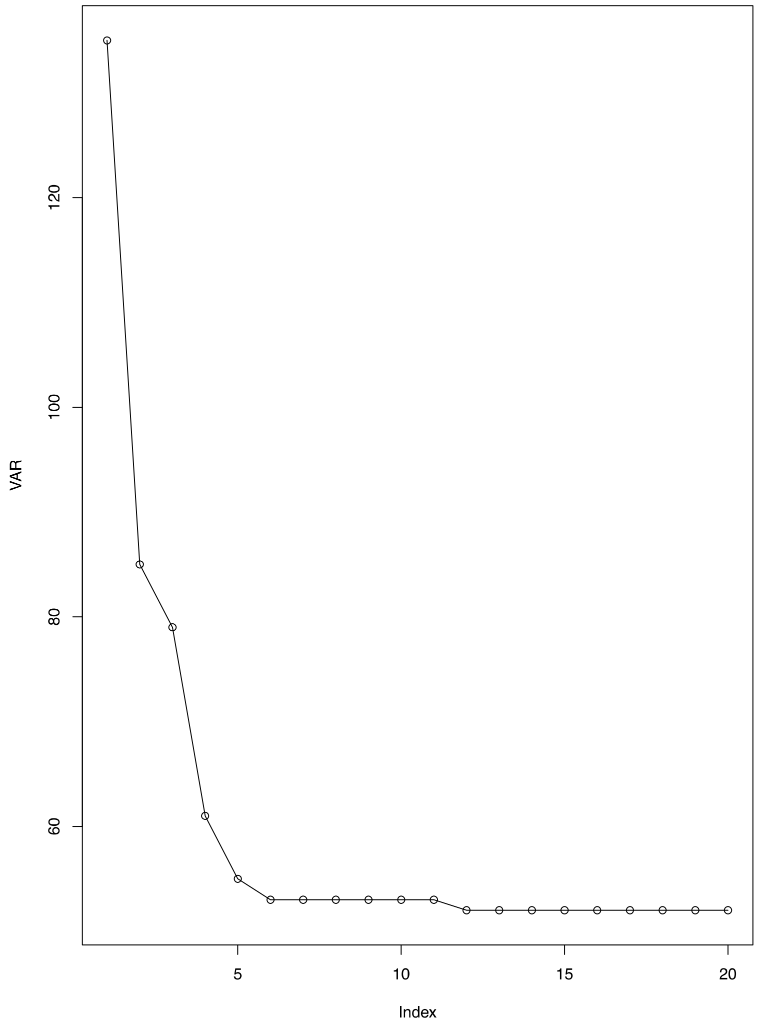

Figure S4. Plot of the coefficient of determination values (r^2) density distribution of random panels test. The green line represents the correlation value obtained for the GW3 panel.

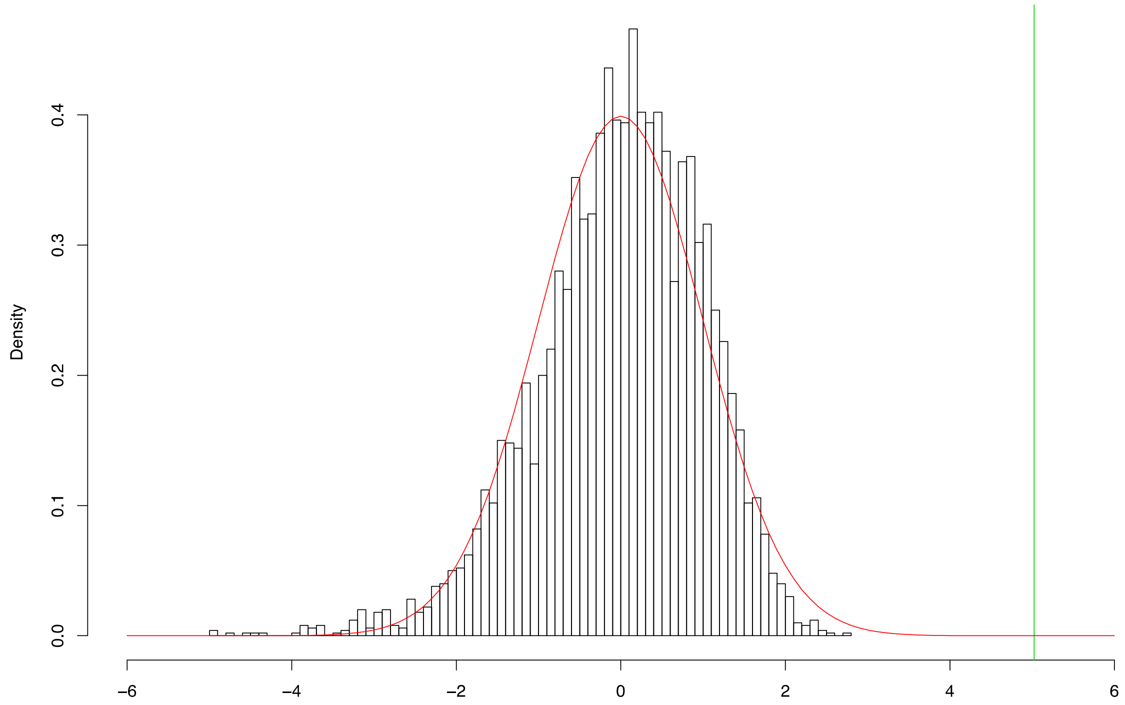

Table S1. Distribution of SNPs per chromosome: for each AIMs panel is reported the exact number of SNPs per chromosome and the percentage related to FS.

	FS	*GW1*	*%*	*GW2*	*%*	*GW3*	*%*	*CH1*	*%*	*CH2*	*%*
1	3760	*126*	*3,35*	*10*	*0,01*	*5*	*0,13*	66	1,75	*3*	0,07
2	3585	*148*	*4,12*	*16*	*0,44*	*7*	*0,19*	73	2,03	*3*	0,08
3	3259	*129*	*3,95*	*15*	*0,46*	*9*	*0,27*	64	1,96	*3*	0,09
4	1765	*77*	*4,36*	*7*	*0,39*	*1*	*0,05*	65	3,68	*3*	0,16
5	1537	*57*	*3,70*	*4*	*0,26*	-	-	46	2,99	*3*	0,19
6	1723	*65*	*3,77*	*11*	*0,63*	*3*	*0,17*	54	3,13	*3*	0,17
7	1504	*49*	*3,25*	*4*	*0,26*	*3*	*0,19*	47	3,12	*3*	0,19
8	1411	*44*	*3,11*	*1*	*0,07*	*1*	*0,07*	41	2,90	*3*	0,21
9	1398	*72*	*5,15*	*6*	*0,42*	*2*	*0,14*	46	3,29	*3*	0,21
10	1151	*43*	*3,73*	*10*	*0,86*	*4*	*0,34*	33	2,86	*3*	0,26
11	686	*27*	*3,93*	*5*	*0,72*	*3*	*0,43*	23	3,35	*3*	0,43
12	1065	*29*	*2,72*	*3*	*0,28*	-	-	31	2,91	*3*	0,28
13	1059	*50*	*4,72*	*6*	*0,56*	*1*	*0,09*	27	2,54	*3*	0,28
14	724	*23*	*3,17*	*2*	*0,27*	*1*	*0,13*	27	3,72	*3*	0,41
15	1057	*50*	*4,73*	*1*	*0,09*	-	-	43	4,06	*3*	0,28
16	996	*38*	*3,81*	*5*	*0,50*	*2*	*0,20*	35	3,51	*3*	0,41
17	885	*34*	*3,84*	*1*	*0,11*	*1*	*0,11*	32	3,61	*3*	0,28
18	891	*32*	*3,59*	*3*	*0,33*	*1*	*0,11*	31	3,47	*3*	0,30
19	739	*39*	*5,27*	*3*	*0,40*	*2*	*0,27*	23	3,11	*3*	0,33
20	715	*31*	*4,33*	*4*	*0,55*	*1*	*0,13*	15	2,09	*3*	0,33
21	546	*11*	*2,01*	-	-	-	-	15	2,74	*3*	0,40
22	678	*29*	*4,27*	*5*	*0,73*	*2*	*0,29*	23	3,39	*3*	0,41
23	679	*20*	*2,94*	*1*	*0,14*	*1*	*0,14*	21	3,09	*3*	0,54
24	443	*20*	*4,51*	*3*	*0,67*	*2*	*0,45*	15	3,38	*3*	0,44
25	639	*18*	*2,81*	*5*	*0,78*	-	-	10	1,56	*3*	0,44
26	586	*18*	*3,07*	-	-	-	-	27	4,60	*3*	0,67

Figure S5. Distribution of SNPs per chromosome in panel CH1.

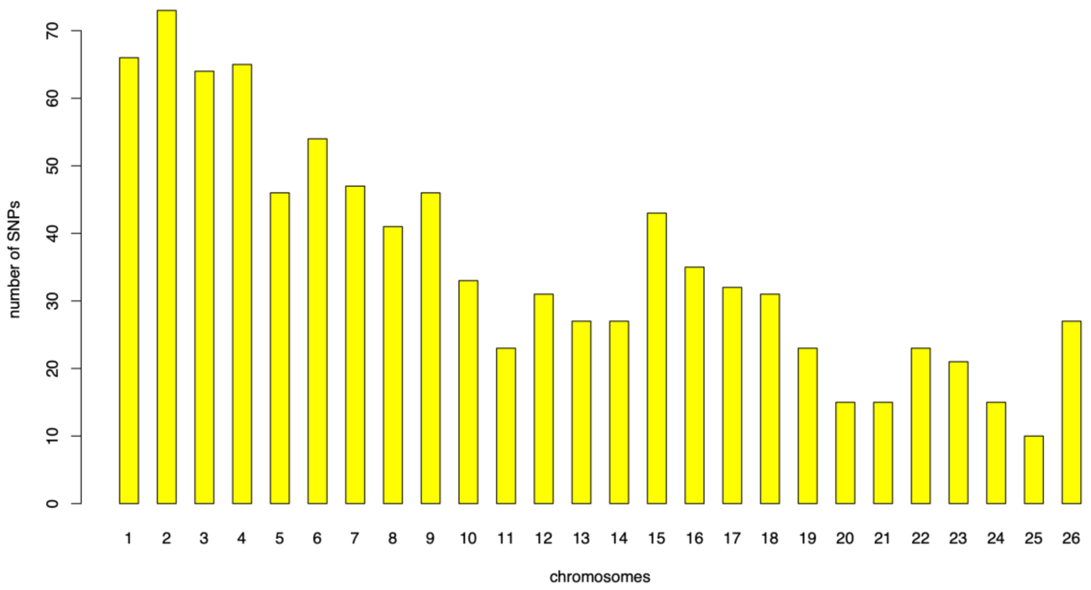

Figure S6. Principal Components Analysis (PC1 vs PC2) analysis and density distribution of mouflon (MSar), hybrid (HYB), and domestic sheep (SAR) populations. The gradient legend on the right side of the plot shows the transition gradient from sheep to mouflon genetic components. The analysis was performed using panel CH1 and CH2.

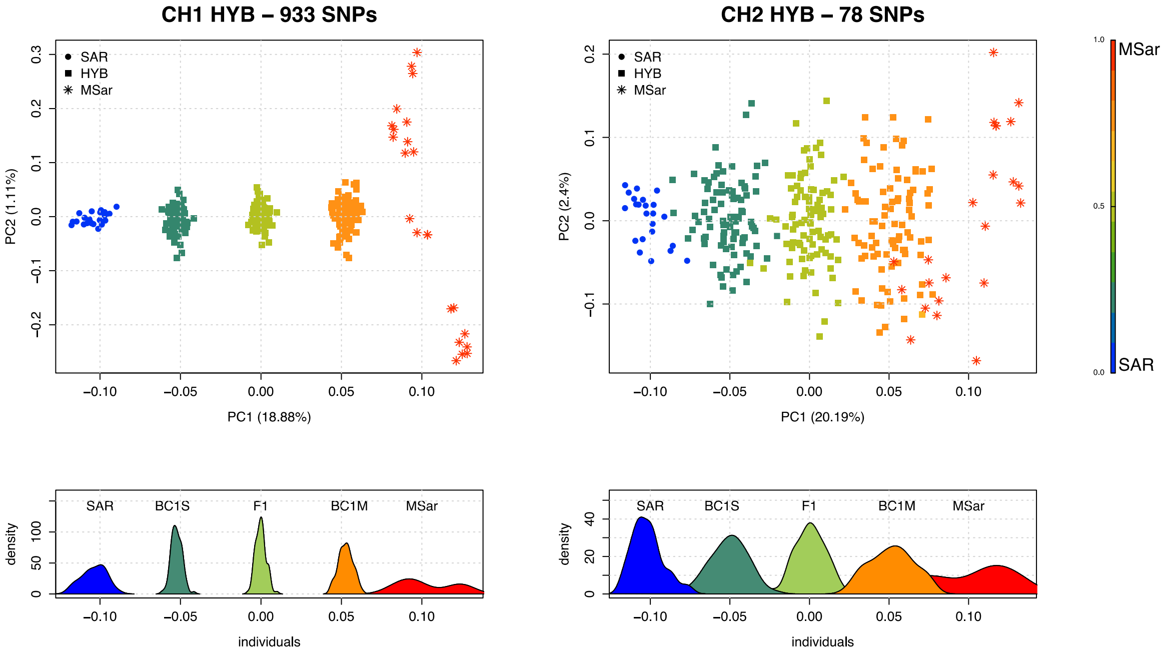

Figure S7. Principal Components Analysis (PC1 vs PC2) analysis and density distribution of mouflon (MSar), mouflon x domestic hybrid (MxS), and domestic sheep (SAR) populations. The gradient legend on the right side of the plot shows the transition gradient from sheep to mouflon genetic components. The analysis was with CH1 and CH2.

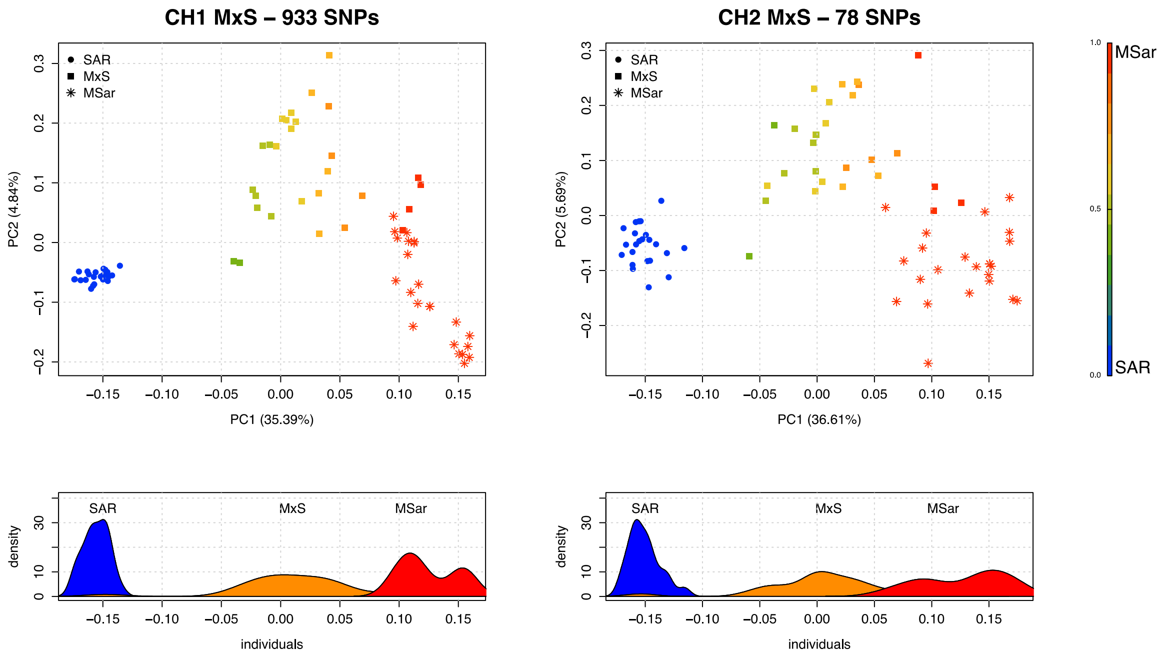

Figure S8. Supervised admixture K=2 plot evaluated with full set on the HYB dataset.

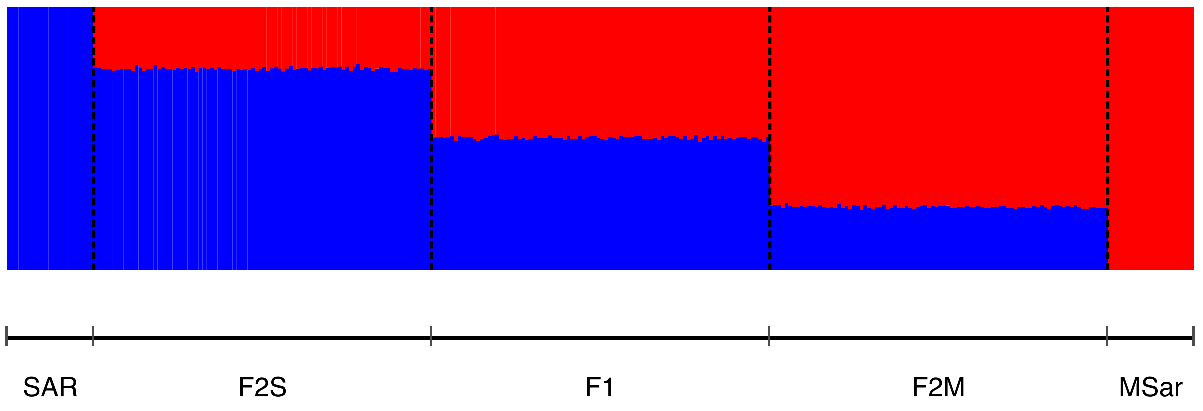

Supplement: Supplementary file 1 [file animals-10-00582-s001.pdf]
